# Supplementary material for: Hallmarks of Hunds coupling in the Mott insulator Ca2RuO4
Source: Nat Commun. 2017 May 5;8:15176. doi: 10.1038/ncomms15176 (PMC5424259; doi:10.1038/ncomms15176)
Supplement: Supplementary Information — Supplementary Figure 1 [file ncomms15176-s1.pdf]

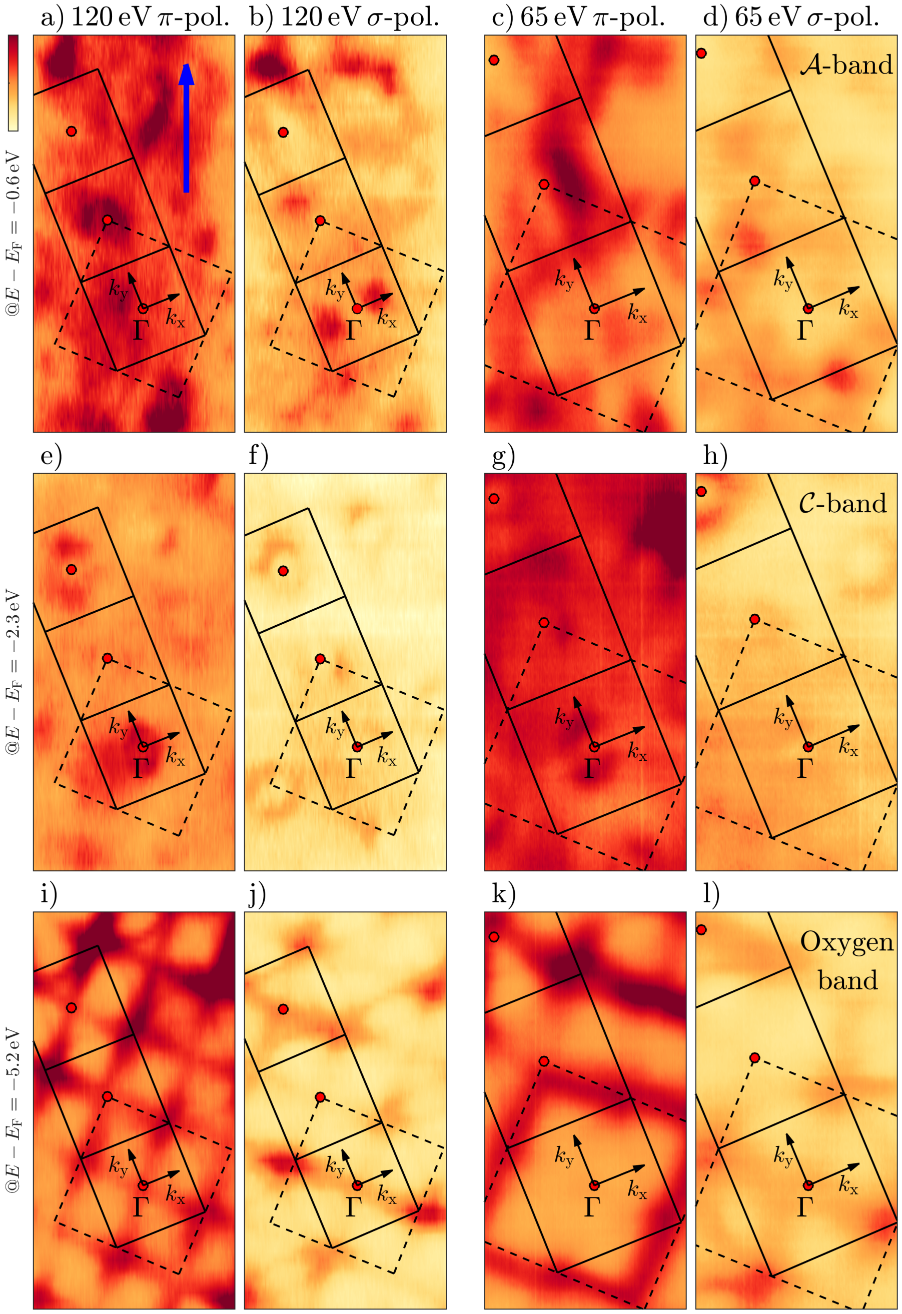

Supplementary Figure 1: **Matrix element effects** ARPES intensity maps of ruthenium and oxygen bands in Ca_2RuO_4 recorded with 120 eV, π-polarised photons in **(a, e, i)**, 120 eV, σ- polarised photons in **(b, f, j)**, 65 eV, π-polarised photons in **(c, g, k)** and 65 eV, σ- polarised photons **(d, h, l)**, respectively. Dark colours correspond to high intensities. Incident direction of the light is indicated by the blue arrow in **(a)**. These data were recorded with an analyser slit oriented with an angle ~30 degrees with respect to the Ru-O bond direction. The orthorhombic zones (boundary of the first zone at $k_x = \pi/a$ and $k_y = \pi/b$, a = 5.39 Å and b = 5.59 Å) are indicated by solid black lines whereas the hypothetical tetragonal zone boundary is displayed with a dashed line. **(a)-(d)** are constant energy maps at binding energy $\varepsilon = E$-E_F = -0.6 eV revealing the matrix element effects of the A-ruthenium band. Notice that the intensities of the A-band is strongest for 65 eV **(c, d). (e)-(h)** are displaying constant energy maps of the C-band (ε = -2.3 eV), clearly observable with 120 eV incident photons **(e, f). (i)-(l)** show intensity maps of the oxygen bands at ε = -5.2 eV.
